# Type 1 Diabetes (T1D) and Latent Autoimmune Diabetes in Adults (LADA): The Difference Between a Honeymoon and a Holiday

**DOI:** 10.1155/2022/9363543

**Published:** 2022-03-18

**Authors:** Livia M. R. Marcon, Carmine G. Fanelli, Riccardo Calafiore

**Affiliations:** Section of Endocrinology and Metabolism, Department of Medicine and Surgery, University of Perugia Medical School, Perugia, Italy

## Abstract

Type 1 diabetes mellitus (T1D) is a chronic autoimmune disease in which destruction of the insulin-producing *β*-cells in the pancreatic islets requires regular lifelong insulin replacement therapy, the only lifesaving treatment available at this time. In young persons with a genetic predisposition, it usually manifests after being exposed to environmental triggers. A subtype of autoimmune diabetes mellitus (ADM) that typically occurs in adulthood is often referred to as latent autoimmune diabetes of adults (LADA). LADA is characterized by a milder process of *β*-cells destruction and less intensive insulin treatment, which may become necessary even many years after diagnosis. Genetic predisposition of T1D carries an increased risk for other autoimmune diseases, such as autoimmune thyroiditis, the most frequently associated condition, and pernicious anaemia (PA), present in approximately 4% of all individuals with T1D. Here, we describe the case of a 90-year-old woman with vitiligo and a mute medical history who was admitted to our University Hospital in Perugia with hyperglycaemia and severe anaemia due to vitamin B12 (VB12) depletion. A short time after setting the beginning treatment with a basal-bolus insulin regimen, her insulin requirement rapidly declined and treatment with sitagliptin, a dipeptidyl peptidase-4 inhibitor (DPP4), was started. A complete autoimmunity screening panel showed that GAD65 and intrinsic factor autoantibodies were positive.

## 1. Introduction

ADM is a chronic autoimmune disease affecting pancreatic insulin-producing *β*-cells that encompasses a wide spectrum of different clinical presentations. T1D is usually diagnosed at a young age; it manifests with ketoacidosis and requires lifelong insulin replacement therapy while LADA emerges later in life and insulin therapy can be postponed for years after diagnosis.

ADM is frequently accompanied by other autoimmune endocrine and nonendocrine diseases, the latter encompassing the so called “autoimmune polyglandular syndromes (APS),” more common in females. Among these is pernicious anaemia (PA), a form of autoimmune gastritis with impaired VB12 absorption.

In this report, we describe the unusual case of a polyautoimmune syndrome clinically emerging at an old age.

## 2. Case Presentation

The patient was a 90-year-old woman admitted to our clinical unit at the Perugia University Hospital, Perugia, Italy, for dyspnoea, anorexia, and agitation. Her body mass index was at the lower normal limit, she had a mute medical history, no family history of diabetes mellitus, and no history of smoking or other unhealthful habits. Relatives did not report previous episodes of anaemia or bleeding or recent viral infections. She was not on medications except furosemide prescribed a week earlier by the family doctor, due to worsening dyspnoea and peripheral oedema in the lower limbs.

The patient tested negative on the SARS-CoV-2 molecular test performed in the emergency department. Blood tests at admission showed a haemoglobin value of 5.4 g/dL with a high mean corpuscular volume (MCV) of 109.1 fl, platelets were 116,000 *μ*L; fasting plasma glucose was 720 mg/dL (40 mmol/L) with a glycated haemoglobin (HbA1c) of 8.4% (68 mmol/mol), underestimated because of the presence of anaemia (Table 1). The arterial blood gas (ABG) analysis showed that the pH was normal (7.41), pCO_2_ was considerably reduced (25 mmHg), plasma osmolarity was equal to 299.8 mmol/kg, and there was a marked increase of the anion gap equal to 19.8 mmol/L, likely due to high lactate levels (6 mmol/L) (Table 2). The latter could likely be explained by different concomitant factors such as severe anaemia-induced hypoxia, red blood cells haemolysis, and glucose toxicity. It has indeed been observed that a condition of hyperglycaemia can be associated with a higher lactate production due to the oversupply of glucose that pushes the glycolytic pathway [1].

Neither blood nor urine ketone bodies were dosed, and considering that the blood pH ranged normal levels, we could rule out with a reasonable degree of certainty the presence of diabetic ketoacidosis (DKA) [2]. Actually, the data did not match the diagnostic criteria of either two hyperglycaemic syndromes, DKA, and hyperosmolar hyperglycaemic state (HHS): we probably intercepted the pathologic process at a time when those diagnostic criteria were unmet.

The physical examination revealed a thin body with hands and face marked with well-defined milky patches of vitiligo. At the rectal examination stools appeared normochromic. Neuropathy was not detected based on clinical history and physical evaluation. Rehydration therapy was immediately started together with subcutaneous administration of fast-acting insulin and blood transfusions. Considering the haemoglobin values compatible with a diagnosis of severe anaemia and the general clinical conditions of the patient, we can assume the anaemia had developed slowly during the months preceding the admission.

Subsequent investigations showed that a process of haemolysis was present: LDH and bilirubin values were high, mostly the indirect form, and haptoglobin was consumed; a faecal occult blood test (FOBT) resulted slightly positive with normal serum iron levels.

We investigated all the major causes of haemolysis: direct and indirect Coombs tests were normal but the VB12 level was largely below the normal range. The presence of severe macrocytic anaemia and haemolysis led us to believe they were both related to VB12 deficiency resulting in ineffective erythropoiesis.

Connecting the dots, we decided to do a complete autoimmunity screening panel and the patient tested positive for GAD65 and intrinsic factor autoantibodies (Table 3). A supplemental therapy with once-weekly VB12 analogue subcutaneous injections was set up together with a basal-bolus insulin regimen. After few days, the daily insulin consumption started to decline until the complete withdrawal.

We concluded the clinical investigations with an abdominal echotomography that excluded hepatic and pancreatic alterations, and with an esophagogastroduodenoscopy that ruled out the presence of bleeding sites but showed chronic gastritis.

The patient was discharged with close outpatient monitoring for one month. At the one-month clinical evaluation, glycaemic control was still acceptable without insulin therapy. In order to prevent hypoglycaemic episodes and preserve the remaining *β*-cells function treatment with daily sitagliptin 100 mg, an oral glucose-lowering drug inhibiting the dipeptidyl peptidase-4 (DPP4) enzyme was started.

The subsequent follow-up visits confirmed the constant fair glycaemic control over time: the blood analysis one year later showed that haemoglobin was 13.7 g/dL, fasting plasma glucose was 92 mg/dL, and glycated haemoglobin was 5.9% (41 mmol/mol). The patient was still on daily sitagliptin 100 mg.

## 3. Discussion

The pathogenesis of T1D is well-known to involve both genetic and environmental factors [3]. Specific human leukocyte antigen (HLA) haplotypes, such as HLA-DR4-DQ8 and HLA-DR3-DQ2 [4], provide the immunological basis of the development of the disease once the subject has been exposed to external antigens. This exposure induces a T-lymphocyte-mediated reaction against pancreatic *β*-cells leading to their destruction and consequently to the loss of insulin production.

Shortly after a T1D diagnosis, a period occurs known as “diabetes honeymoon phase,” or remission phase, lasting a variable length of time, usually from weeks to months; nevertheless, there have been some reported cases of this honeymoon lasting for years [5, 6].

The hallmarks of the honeymoon period are low or no insulin requirement together with a fair blood glucose control, with less glucose variability, less risk of hypoglycaemia, and lower overall average blood glucose levels [7]. In this phase, the remaining insulin-producing cells keep on working until they are finally killed off and the honeymoon comes to an end with insulin needs rising again.

In the case of LADA, the destruction of the insulin-producing cells by the self-reactive T lymphocytes proceeds at a slower speed and more mildly. Although questioned by some authors [8, 9], the criteria on which the diagnosis usually rests were proposed by the Immunology of Diabetes Society (IDS) in 2005: (1) onset age greater than 35 years, (2) islet autoantibodies as a marker of the autoimmune process, and (3) insulin independence for at least 6 months after diagnosis [10]. LADA shares genetic susceptibility and clinical phenotype both with T1D and T2D, thus suggesting it is a continuum between the two extremes [11].

Compared to patients with isolated T1D, those with T1D plus autoimmune diseases (AIDs) are older and exhibit a higher female: male ratio; average patient age and age at disease onset are higher in T1D plus AID vs T1D only [12]. Disease risk is associated with organ-specific autoantibodies, which can be used to screen subjects with T1D [13, 14]. Among these diseases, hypothyroidism occurs most frequently while adrenal gland insufficiency occurs the least. LADA has wide genetic overlap with T1D, actually sharing a great risk of other AID. Moreover, the risk seems to be related to the GAD65 titre displayed by the subject at the moment of diagnosis [15], and, as in T1D, is maximum for hypothyroidism and minimum for adrenal gland insufficiency [15, 16].

In particular, in people with T1D, PA has a mean prevalence of 4.3% vs 0.2% in the general population [17], while no data are reported in the literature about the prevalence of PA in subjects with LADA. PA is marked by the presence of circulating antibodies to intrinsic factor which prevent VB12 absorption leading to a progressive decrease in vitamin storage, which is ten times more common in people with T1D than in nondiabetic persons [18, 19]. VB12 deficiency, in turn, leads to megaloblastic anaemia and neurological symptoms such as peripheral neuropathy and cerebral manifestations (confusion and psychosis) [20].

A Japanese report of people of all ages and both genders with concomitant PA and T1D showed that the subjects were mostly older women (the oldest was 87 years old) who exhibited T1D approximately 10 years prior to PA and had other AID, especially thyroiditis [21]. In the case of our 90-year-old female patient the association of vitiligo, ADM and PA matches the criteria of APS-4.

Differential diagnosis between T1D and LADA can sometimes be presumptive because rather than totally different pathological entities, they represent a seamless continuum. When the patient was initially referred to our clinical unit, we thought she could have T1D due to her phenotype (thin and with vitiligo) and the concomitant acute occurrence of another AID, PA. When her insulin needs declined so quickly until the complete withdrawal, we wondered whether it was a honeymoon phase of T1D or a LADA, the two autoimmune conditions under which the insulin secretory capacity is preserved. The marked improvement in glycaemic control on sitagliptin treatment alone during the following months confirmed the second hypothesis and fully fulfilled the IDS LADA criteria [10].

After carrying out a comprehensive review of the currently available literature, we found no cases of APS diagnosed above 90 years old and only few cases of autoimmune diabetes emerged in very elderly individuals. Oriot et al. [22] reported the case of a 93-year-old woman whose diabetes was undoubtedly T1D as the onset was characterized by ketoacidosis proven by the presence of high levels of ketones in the urine sample together with metabolic acidosis. Moreover, the woman tested positive for more than one pancreatic islet autoantibody and immediately after the diagnosis insulin therapy became an irreplaceable treatment. A case of T1D in a 93-year-old woman with very akin clinical features has now been reported also by Ahmad et al. [23]. The oldest ultraelderly case of acute-onset autoimmune diabetes described so far by Yamaguchi et al. is instead of a 96-year-old Japanese woman who presented without ketoacidosis and in which insulin therapy became pleonastic few days after the diagnosis despite high anti-GAD antibody titre. She died few months after the discharge and whether she was experiencing a honeymoon phase or a LADA was impossible to ascertain [24].

The paucity of cases reporting newly diagnosed ADM in the elderly underlines the exceptionality of our case but could also be a warning of the risk of misclassification of adult-onset diabetes. Actually, there may be a latent proportion of adult-onset ADM misclassified as T2D due to the heterogeneous clinical features of LADA. Moreover, as the human average lifespan is lengthening, acute-onset autoimmune diabetes at an advanced age may be increasingly frequent in the close future.

In accordance with the current literature, our report demonstrates that a person may be born with a genetic pattern predisposed to autoimmunity, but this is not enough to make the disease surface. The time may come, however, when due to an external trigger such as an infection, the immune system reacts and misrecognizes its own genetic epitopes destroying the cells on which they are expressed. Contrary to the Japanese report cited above [21], we can see looking at our patient's laboratory data from the years before she came to our attention, VB12 depletion and the progressive increase in fasting plasma glucose began roughly at the same time. In 2013, seven years before the admission, fasting plasma glucose was slightly increased and VB12 was at the lower limit of normal with a haemoglobin amount related to the age of the patient, while in 2019, the year before admission, glycemia was far beyond a normal range and the VB12 pool was declining, but still no anaemia appeared. As far as we know, no treatment was initiated to correct both VB12 depletion and hyperglycaemia.

It is also important to underline that being a genetic predisposition the *sine qua non* for AID emergence, there is a high prevalence of autoimmunity recurrence in offspring. In fact, both sons of the patient have vitiligo: we invited them to perform a complete autoimmunity screening panel and to maintain longitudinal control with regular blood tests.

## Figures and Tables

**Table 1:** Laboratory data.

Variable	Reference range, adults	Exams 7 years before admission	Exams 1 year before admission	Exams on admission	Exams at discharge	Exams 1 year after discharge
Haemoglobin (g/dL)	12–16	13	12.6	5.4	10	13.7
Haematocrit (%)	37–47	40.6	38.9	15.5	30	41.9
MCV (fl)	82–97	80.4	85.9	109.2	90.4	83.1
White cell count (per *μ*L)	3,600–9,600	7,750	7,100	5,300	9,240	5,900
Platelets (*μ*L)	140,000–440,000	382,000	340,000	116,000	650,000	248,000
Sodium (mEq/L)	136–146	143	139	130	145	142
Potassium (mEq/L)	3.5–5.1	4.5	4.5	4.4	3.4	4.6
Chloride (mEq/L)	101–109			102	111	
Urea nitrogen (mg/dL)	17–43	41	37	61	28	51
Creatinine (mg/dL)	0.55–1.12	0.57	0.71	0.80	0.89	0.87
Fasting blood	60–99	118	154	720		92
Glucose (mg/dL) (mmol/L)	3.9–5.5	6.55	8.55	40		5.1
HbA1c (%) (mmol/mol)	4.3–5.9 23–41		8.4 68			5.96 42
Cholesterolemia (mg/dL)	70–190	258	246			240
Triglycerides (mg/dL)	<180	158	188			122
HDL cholesterol (mg/dL)	>40	82	70			56
Total bilirubin (mg/dL)	0.30–1.20	0.67	0.60	2	0.86	
Direct bilirubin (mg/dL)	0.00–0.20			0.43		
Alanine transaminase (UI/L)	0–35	13	9	9	5	7
Aspartate transaminase (UI/L)	0–35	16	17	13	11	13
Haptoglobine (mg/dL)	30–200			1	111	
Sideremia (mcg/dL)	60–180	90		207		
25-Hydroxyvitamin D (ng/mL)	>20		9.8	23.8		
Vitamin B12 (pg/mL)	180–914	203	160	44	>1,500	192
Folate (ng/mL)	3–20		10.3	5	>24.7	
TSH (uUI/mL)	0.34–5.60		1.250	1.180		

**Table 2:** Arterial blood gas analysis.

Variable	Reference range	On admission
pH	7.35–7.45	7.41
pCO_2_ (mmHg)	35–45	25.7
pO_2_ (mmHg)	75–100	70.4
HCO_3_-std (mmol/L)	21–27	18.2
mOsm (mmol/kg)	275–295	299.8
AG (mmol/L)	4–12	19.8
Fasting blood glucose (mg/dL)	60–99	722
Lactate (mmol/L)	0.4–0.8	6

**Table 3:** Autoimmunity panel.

Variable	Reference range	Patient's value
IF Ab (U/ml)	<6	73.5
Anti-GAD65 Ab (U/ml)	<5	>250
21-OH Ab (U/ml)	<0.4	0.13
Parietal cell Ab	<1 : 140	<1 : 140
TPO Ab (UI/ml)	0–9	0.2
Tissue transglutaminase-IgA Ab (U/ml)^§^	<10.0	0.40

^§^Total IgA = 225 mg/dL. IF: intrinsic factor.

## Data Availability

The data are available in the archives of our hospital.
